# Assessing gut barrier integrity in ruminants: structural, functional, and immunological approaches

**DOI:** 10.1093/af/vfag011

**Published:** 2026-05-08

**Authors:** Jon P Schoonmaker

**Affiliations:** Department of Animal Science, Purdue University, West Lafayette, IN

**Keywords:** Barrier integrity, Gastrointestinal tract, Inflammation, Ruminant

Implications

- Gastrointestinal barrier dysfunction in ruminants imposes substantial metabolic costs, diverting nutrients away from growth and production and elevating susceptibility to inflammation and disease.
- Targeted pharmacological models (e.g., aspirin, indomethacin, gamma-secretase inhibitors) offer controlled ways to study epithelial injury but differ from real-world stressors.
- Common production stressors—such as transportation, heat stress, feed restriction, and high-grain diets—compromise barrier function and can serve as effective research models because they closely replicate real-world field conditions.
- Accurate assessment of gut barrier integrity requires integrating functional permeability assays with histology, tight junction evaluation, and systemic inflammatory biomarkers, as no single method provides a complete picture.
- Advancing our understanding of ruminant gut health will depend on refining existing models, validating non-invasive biomarkers, and developing field-ready diagnostics that link epithelial biology to meaningful outcomes in cattle health and productivity.

The gastrointestinal tract (GIT) of ruminants represents one of the most complex and energetically demanding organ systems in the animal body. It functions not only as the site of digestion and nutrient absorption but also as a critical immune barrier, housing approximately 70% of the body’s total immune tissue (Vighi et al., 2008). The metabolic requirements of maintaining this vast immune interface are substantial, with estimates suggesting that nearly 20% of oxygen consumption and 30% of whole-body protein synthesis are devoted to gut activity (McBride and Kelly, 1990).

Gut barrier integrity is frequently challenged by environmental and management stressors. Transportation, heat stress, dietary transitions, and feed restriction are among the most common conditions that disrupt gut homeostasis in ruminants. These stressors trigger neuroendocrine responses, alter feed intake and microbial populations, and promote the release of bacterial endotoxins such as lipopolysaccharide (LPS). The consequence is often an inflammatory cascade that compromises epithelial integrity and increases intestinal permeability. Chronic or unresolved inflammation imposes substantial energetic costs on the animal redirecting nutrient away from lean tissue gain or lactation toward immune activation.

Given these challenges, reliable methods to evaluate gut health and barrier function are vital for advancing ruminant research and guiding the development of targeted therapies. Over the past several decades, researchers have developed a range of approaches to assess morphological changes in gut tissue, measure molecular markers of inflammation, and quantify permeability using orally administered probes.

## Structure and Function of the Ruminant GIT

**Figure 1.**
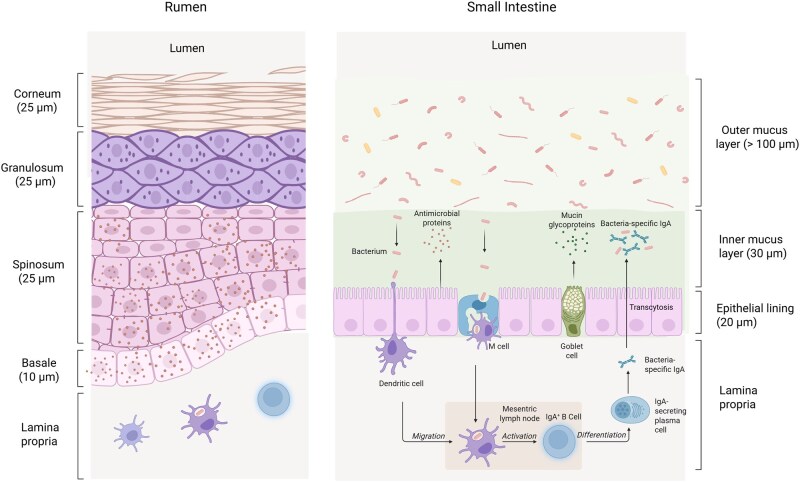
Ruminal and intestinal barrier structure. Image created using bioRender.

The ruminant GIT is uniquely adapted to manage the challenges of a microbial fermentation system in the rumen followed by enzymatic digestion in the small intestine (Figure 1). The reticulo-rumen and omasal epithelia, lined with 4 layers of stratified squamous cells, are able to withstand abrasive plant material and tolerate dense microbial populations, while simultaneously absorbing small molecules such as volatile fatty acids and ammonia (Steele et al., 2016). Post-omasal regions are characterized by a single-layered epithelium, embedded with specialized and organized immune cells, protected by a multi-layered mucus barrier that facilitates nutrient uptake while limiting movement of pathogens (Steele et al., 2016). Together, these regions balance nutrient assimilation, tolerance of commensal microbes, and defense against invading organisms.

### Rumen

Gene expression studies have shown that ruminal epithelial cells share similarities with keratinized tissues like the skin and esophagus (Chen et al., 2019), organs typically associated with protective functions rather than absorption (Baldwin and Connor, 2017). The reticulo-rumen and omasal epithelium is stratified, with the continuously shedding stratum corneum providing mechanical protection against abrasive feed particles and dense microbial populations (Xiang et al., 2016). Beneath this cornified layer, the stratum granulosum, stratum spinosum, and stratum basale support limited absorptive capacity, permitting passage of only small, water-soluble molecules such as volatile fatty acids and ammonia (Garcia et al., 2017). Tight junction proteins (e.g., claudins, occludins, and zonula occludens) are absent from the stratum corneum and are primarily located in the stratum granulosum and stratum spinosum, where they regulate paracellular transport and contribute to barrier integrity (Baldwin and Connor, 2017). However, expression patterns within the different strata vary by protein, and their functional contribution to barrier regulation is less pronounced than in the intestinal epithelium (Graham and Simmons, 2005; Stumpff et al., 2011). The ruminal lamina propria contains a dispersed population of immune cells such as macrophages, dendritic cells, and B and T lymphocytes but lacks the specialized immune cell subsets and organized lymphoid tissues characteristic of the intestine (Garcia et al., 2017).

### Intestine

The intestinal epithelium is composed of a single layer of columnar cells interspersed with specialized secretory and immune-related cells, including goblet cells, Paneth cells, enteroendocrine cells, and M cells. Villi project into the luminal contents and are contiguous with glands located at the base of the villi: the crypts of Lieberkuhn, or intestinal crypts. Adjacent epithelial cells are connected by tight junction proteins (e.g., claudins, occludins, and zonula occludens) that regulate paracellular permeability and maintain barrier integrity. Goblet cells produce mucus, which forms a protective layer that separates luminal microbes from the epithelium while still permitting efficient absorption of amino acids, sugars, vitamins, and minerals. Beneath the epithelial layer, the lamina propria contains organized lymphoid structures such as Peyer’s patches, along with a dense network of immune cells collectively referred to as gut-associated lymphoid tissue (GALT), which provides immunological surveillance and defense. Importantly, recent work has shown that approximately 60% of total gastrointestinal leakiness in ruminants originates from the lower GIT (Bertens et al., 2024), underscoring the small and large intestines as major sites of barrier dysfunction under stress.

## Barrier Function Mechanisms/Physical Barriers

### Mucus

The mucus layer is a critical component of the gastrointestinal barrier, serving as the first line of defense against physical, chemical, and microbial insults. Mucus is categorized into two structurally distinct layers (Figure 1), a loose adherent outer layer containing microbes and a firmly attached inner layer mostly devoid of microbes (Steele et al., 2016). Secreted primarily by goblet cells, mucus consists of 95% water with the remaining 5% composed largely of mucin glycoproteins that form a gel-like protective coating over the epithelium. It acts as both a lubricant to reduce mechanical abrasion and a biochemical shield that limits microbial adhesion and invasion (Johansson et al., 2013). Mucus also provides a matrix to keep antimicrobial peptides and immunoglobulins in a strategic location, enhancing mucosal defense. Mucus characteristics in ruminants have not been described as they have for non-ruminants where the inner and outer mucus layers in the rat measure approximately 15–30 and 100–450 μm in the small intestine and 75–150 and 600–800 μm in the colon, respectively (Atuma et al., 2001). Goblet cells, distributed along the intestinal and colonic epithelium, dynamically respond to dietary and microbial cues. An increase in the number of goblet cells, or hypertrophy of existing cells, is often observed under conditions of short term stress or inflammation (Kvidera et al., 2017b), reflecting the host’s effort to reinforce the mucosal barrier. Conversely, a reduction in the number of goblet cells or mucin content may signal impaired barrier function and heightened susceptibility to pathogen invasion (Jung and Saif, 2017). Thus, quantifying goblet cell density and activity is an important histological measure of gut health in ruminant research. Frequent observation of mucin casts, visible in feces as sloughed mucus structures, suggests chronic epithelial irritation and compromised barrier function, which are linked to reduced nutrient absorption and impaired animal performance.

### Cell turnover

An essential aspect of gastrointestinal barrier defense is the continuous renewal of epithelial cells. Both the rumen and intestinal epithelia are dynamic tissues, in which cell turnover helps maintain structural integrity, remove damaged cells, and limit microbial access to the underlying lamina propria (Williams et al., 2015). This process ensures that the epithelium remains both absorptive and protective under the constant challenges of digestion, microbial activity, and dietary variation.

Intestinal epithelial cells originate in the crypts of Lieberkühn and migrate toward the villus tip where they are shed into the lumen. This continuous renewal, with a turnover time of only 2–6 days in most mammals, maintains epithelial integrity and limits microbial access to underlying tissues (Mayhew et al., 1999). Two common morphometric indicators of intestinal health are villus height and crypt depth. Greater villus height corresponds to a larger absorptive surface area, reflecting efficient nutrient assimilation and a robust epithelial barrier (Williams et al., 2015). Conversely, deeper crypts often signal increased epithelial turnover in response to injury or inflammation (Williams et al., 2015). Crypt hyperplasia is energetically costly, as more nutrients are diverted to epithelial regeneration, and is frequently associated with villus atrophy (Williams et al., 2015). Thus, an ideal morphology is characterized by tall villi and shallow crypts, maximizing absorption while minimizing unnecessary cell proliferation. Alterations in villus height-to-crypt depth ratios are frequently used as biomarkers of gut barrier dysfunction in both ruminant and non-ruminant species. However, much of the microscopic work in ruminants has limited biological and technical replication, and methodologies used to measure key criteria are often not clearly described. Intestinal mucosa is highly susceptible to post-mortem autolysis and mechanical damage, which can distort villus–crypt architecture and introduce measurement artifacts. Prompt collection after slaughter, gentle handling of intestinal tissue, careful removal of luminal contents, and rapid fixation are therefore critical for preserving epithelial structure and ensuring reliable morphometric measurements.

In the rumen, epithelial renewal occurs through sloughing of the outer stratum corneum and regeneration from basal layers, helping remove damaged cells and limit microbial attachment (Graham and Simmons, 2005; Baldwin and Connor, 2017). The turnover time for ruminal epithelium is 4 to 17 days, with diet influencing the rate: 11 days for grain-fed cattle, 17 days for forage-fed cattle, and 4 days for transitioning cattle (Goodlad, 1981).

The importance of cell turnover becomes most evident under stress. During heat stress, nutrient restriction, or acidosis, cell turnover rates throughout the GIT are disrupted. These disruptions may include sloughing of papillae, rumenitis, and thinning of the epithelium in the rumen (Owens et al., 1998; Pederzolli et al., 2018) as well as villus shortening and crypt hyperplasia in intestinal regions (Tao et al., 2014; Kvidera et al., 2017a; Yu et al., 2025). Such changes compromise absorptive capacity and increase the likelihood of barrier failure, allowing microbial products such as lipopolysaccharide (LPS) to enter circulation.

## Transport Pathways Across the Ruminant Intestinal Epithelium

**Figure 2.**
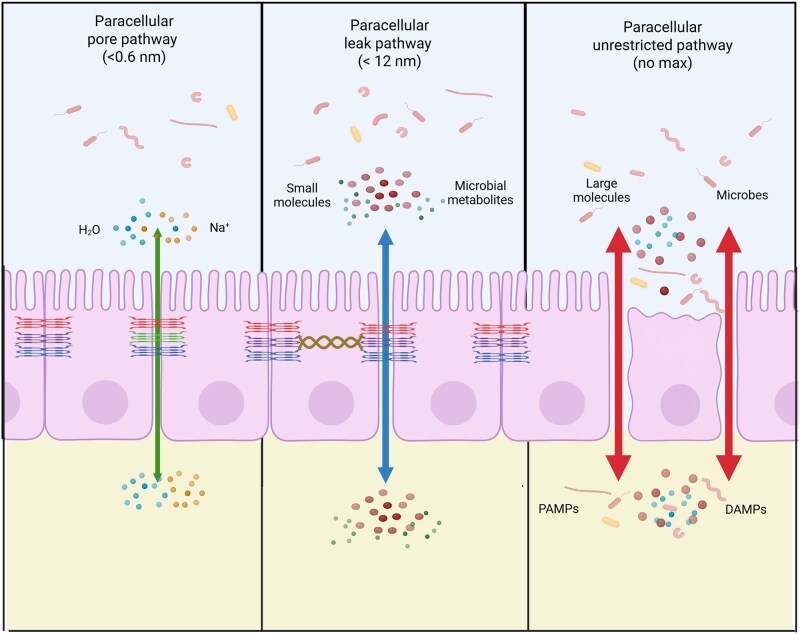
Intestinal paracellular permeability. Three pathways contribute to paracellular transport: a high-conductance, charge- and size-selective pore pathway (green), a low-conductance, size-selective leak pathway (blue), and a nonselective unrestricted pathway (red). The pore and leak pathways are regulated by tight junctions, the unrestricted pathway is not. The leak pathway can be activated by physiological or inflammatory stimuli, while the unrestricted pathway arises at sites of epithelial damage, allowing both small and large molecules, including intact microbes, to access the lamina propria. DAMP, damage-associated molecular patterns. PAMP, pathogen-associated molecular patterns. Image created using bioRender.

**Figure 3.**
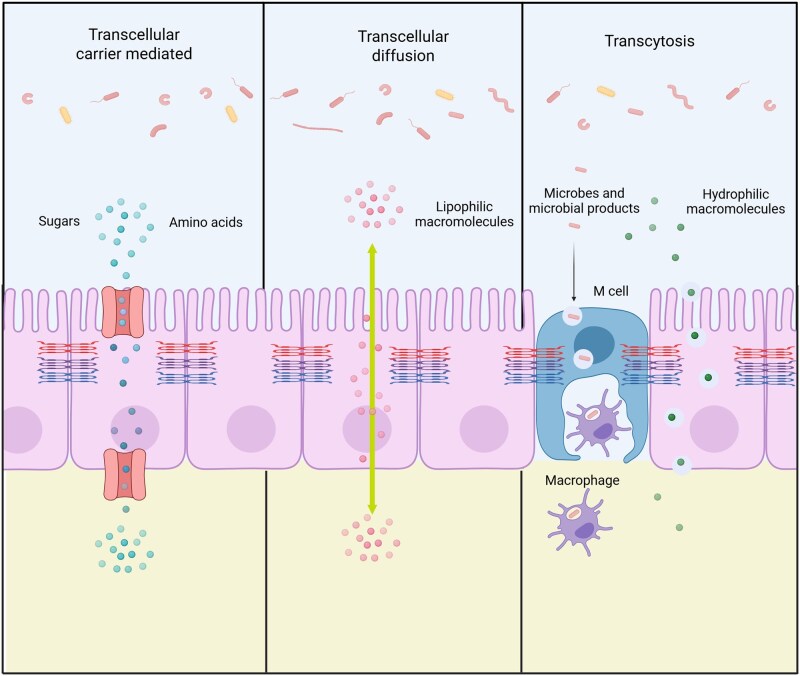
Intestinal transcellular permeability. Transcellular transport occurs through three mechanisms: carrier-mediated transport (facilitated diffusion), passive diffusion, and transcytosis. Large hydrophilic molecules, such as sugars and amino acids, are absorbed via carrier-mediated transport through membrane transporters with high substrate specificity. Lipophilic compounds typically cross epithelial membranes by passive diffusion. Larger antigens and microbial products can be transported across the epithelium by transcytosis, in which specialized M cells internalize luminal material by endocytosis and deliver it to underlying immune tissues. Some pathogens exploit the transcytosis pathway to invade host tissues. Image created using bioRender.

Compounds cross the intestinal epithelium of ruminants primarily through two routes: paracellular transport between adjacent cells (Figure 2) and transcellular transport through epithelial cells (Figure 3). A third permeability route, the unrestricted pathway, is created by epithelial damage; it is tight junction independent, high capacity, and non-selective (Horowitz et al., 2023). Understanding these pathways provides the context needed to interpret permeability measurements and explains why particular methods are used. Transcellular transport encompasses mechanisms that move solutes directly through epithelial cells: carrier mediated transport, diffusion, and transcytosis. Paracellular transport is less selective and allows molecules to pass between adjacent epithelial cells via the pore pathway or the leak pathway (Horowitz et al., 2023). Both pore and leak pathways are size-selective, and the pore pathway is charge selective. Paracellular transport across the pore and leak pathways are regulated by tight junction complexes that link epithelial cells. These multiprotein complexes form a selective seal that in healthy gut tissue permits mainly small, hydrophilic molecules such as water and electrolytes to diffuse in between epithelial cells (Horowitz et al., 2023).

## Measuring Permeability

Assessing permeability in ruminants requires methods that capture both structure and function of the intestinal barrier. Two widely used approaches are marker probe assays and tight junction (TJ) protein analysis. Marker probe assays provide a direct, functional measure of barrier leakiness by tracking the movement of non-metabolized compounds across the epithelium into blood or urine and encompasses paracellular and unrestricted leak pathways. In contrast, evaluation of TJ proteins—through expression levels, localization, or distribution—offers mechanistic insight into how epithelial cells regulate paracellular transport. Together, these complementary methods allow researchers to link functional outcomes with underlying molecular changes in gut barrier integrity.

### Tight junction gene expression

Acute stress or the onset of a challenge can transiently increase tight junction protein gene expression, reflecting activation of early defensive and repair pathways. However, tight junction integrity is regulated primarily through post-translational mechanisms, so mRNA abundance alone is often a poor predictor of functional barrier integrity. In many ruminant models, increases in tight junction transcripts may occur within hours to a few days (McCann et al., 2016; Pederzolli et al., 2018; Briggs et al., 2020), potentially reflecting compensatory or protective signaling rather than improved barrier integrity. Permeability can still be elevated during this phase. Chronic inflammation or sustained barrier injury, however, tends to result in downregulation or mislocalization of these proteins (Hu et al., 2022; Lai et al., 2022) and structural changes such as villus atrophy and crypt hyperplasia may develop. This later phase is more consistently associated with persistent leakiness rather than adaptive compensation. However, the temporal progression of intestinal permeability changes in cattle remains incompletely defined. Sampling within the first 24–72 hours of a stressor may capture a protective compensatory transcriptional response, whereas later sampling (e.g., ≥ 5–14 days, depending on the challenge) is more likely to reflect sustained epithelial dysfunction. Because tight junction proteins function as part of a continuous belt-like junctional complex, assessing their localization by immunostaining would provide spatial context that may offer more informative evidence about recent epithelial stress and repair, even from a single time-point sample.

### Marker probes

Administering inert, non-metabolized probes and quantifying their appearance in blood or urine is a practical way to obtain a functional readout of epithelial leakiness in vivo (Table 1). This strategy integrates the combined effects of tight-junction status, epithelial turnover, mucus, and luminal environment, and they help interpret molecular measurements (e.g., tight junction gene/protein expression) in terms of actual permeability.

**Table 1.** Marker probes used to assess in vivo gastrointestinal tract permeability

Marker	Pore size diameter, Å	Pathway	Sampling	Analysis	Comments
Cr-EDTA*^a^*	8–10	Paracellular leak	Urine & Blood	AA (> 1 mg/kg), ICP (< 1 mg/kg)	No microbial degradation.
Co-EDTA	8–10	Paracellular leak	Urine & Blood	AA (> 1 mg/kg), ICP (< 1 mg/kg)	No microbial degradation. Can dissociate under reducing conditions similar to the rumen
Fluorescin	8–10	Paracellular leak	Urine & Blood	Fluorometric	Microbial degradation. pH sensitive. Binds to some proteins.
Iohexol	12–16	Paracellular leak	Urine & Blood	HPLC, MS, ICP	No microbial degradation.
Lactulose/Mannitol	8–10/6–8	Paracellular leak/Paracellular pore	Urine & Blood	HPLC, GC, MS, enzymatic assay	Rapidly fermented.
Polyethylene glycol (PEG)	8–70, depending on molecular weight	Paracellular leak/Unrestricted	Blood	HPLC, GC, MS, or colorimetric	Some microbial degradation of low molecular weight forms. Wide range of diameter. Preparations may not be uniform in size. Molecular size makes it much more sensitive to kidney filtration dynamics. Will bind tannins.
FITC*^b^*-dextran	28–36	Unrestricted	Blood	Fluorometric	Microbial degradation risk. Molecular size makes it much more sensitive to kidney filtration dynamics. pH sensitive. Costly.

a EDTA = Ethylenediaminetetraacetic acid.

b FITC = Fluorescein isothiocyanate.

In non-ruminants, paired sugar tests (e.g., lactulose: mannitol) are common. In pre-ruminant calves, before the rumen is fully functional, probes susceptible to microbial degradation (lactulose, mannitol, FITC-dextran, fluorescein) have been used much like in non-ruminants (Duff et al., 2020; Pisoni and Relling, 2020). In functional ruminants, however, ruminal fermentation can degrade carbohydrate probes, reducing interpretability. For this reason, metal–EDTA chelates—most commonly chromium-EDTA (Cr-EDTA) and cobalt-EDTA (Co-EDTA)—are preferred because they resist microbial metabolism, remain soluble, and move primarily through paracellular pathways (Bertens et al., 2024).

A common approach in cattle is a single oral bolus of Cr- or Co-EDTA, followed by serial sampling for 24–48 h (Bertens et al., 2024). Probes can be measured in blood or urine. Blood yields pharmacokinetic profiles, but concentrations are low, necessitating sensitive analytics. Urine concentrates the marker, enabling fractional dose recovery measurements with less sensitive analytics. However, obtaining accurate cumulative recovery usually requires indwelling catheterization to allow timed, quantitative collections. Catheters themselves can irritate the animal, and results may be confounded by differences in renal clearance related to hydration status or kidney function. When urinary catheters are impractical, timed coccygeal venipuncture can substitute provided sensitive analytics are available (Bertens et al., 2024).

When marker probes are administered to cattle on diets differing in forage-to-concentrate ratio, or if intake is limited in some way, digesta passage rate can influence the timing of probe appearance in urine. Faster passage may shorten the lag between dosing and marker excretion, whereas slower digesta transit delays urinary recovery. Thus, rate of gastrointestinal passage may need to be accounted for when diet or intake are altered or used as a GIT stress model. Traditional ruminant nutrition techniques may help address this challenge, including the use of cannulated animals and controlled or continuous infusion to standardize probe delivery and better account for passage dynamics. Cannulation further allows probes to be dosed ruminally or post-ruminally, enabling more precise evaluation of regional permeability (Bertens et al., 2024). Future work should also focus on developing marker probes capable of capturing multiple leak pathways. While simple sugars are unsuitable in mature ruminants due to microbial degradation in the rumen, compounds such as polyethylene glycol may be useful because it is inert and available in multiple molecular sizes.

## Immune Sampling and Inflammatory Markers

Immune surveillance by intestinal M cells and dendritic cells involves sampling luminal contents and transporting high–molecular weight antigens across the epithelium via transcytosis, a process in which antigens are internalized by endocytosis and delivered to the lamina propria for presentation to B and T lymphocytes, thereby initiating adaptive immunity (Chase and Kaushik, 2019). While normally protective, this immune surveillance pathway can be exploited during stress when the immune system is overactivated, resulting in greater endotoxin entry and systemic inflammation.

Measuring blood concentrations of cytokines, generated at the site of barrier disruption, and acute phase proteins, produced in the liver, offer an indirect yet informative measure of immune inflammation and gut barrier dysfunction, reflecting the systemic response to luminal leakage. When the gastrointestinal environment becomes disrupted—such as during microbial lysis or tissue damage—both microbial products and host-derived stress signals activate the immune system. Microbial-associated molecular patterns (MAMPs), which are structural components essential for microbial survival, and damage-associated molecular patterns (DAMPs), which are molecules released from host cells in response to injury or stress, act as the primary triggers of this response (Garcia et al., 2017).

Recognition of MAMP and DAMP by pattern recognition receptors (PRR), including toll-like receptors (TLR), activates inflammatory signaling pathways such as NF-κB, leading to cytokine production, recruitment of immune cells, and initiation of the acute phase response (Garcia et al., 2017). Cytokines recruit innate immune cells (neutrophils, monocytes, and macrophages) to sites of barrier injury, amplify local inflammation, and stimulate the liver to initiate the acute phase response. By loosening epithelial junctions, cytokines also increase permeability to facilitate immune cell trafficking. Recruited innate immune cells secrete antimicrobial peptides and eliminate engulfed pathogens via reactive oxygen species and proteolytic enzymes within phagolysosomes. When activation becomes excessive or prolonged, extracellular release of these mediators can injure epithelial cells, compromise tight junction integrity, and exacerbate barrier permeability. In addition to structural consequences, this inflammatory response imposes a substantial metabolic cost; for example, Kvidera et al. (2017a) reported that acute activation of the innate immune system induced by lipopolysaccharide challenge in mature Holstein dairy cows consumed more than 1 kg of glucose over a 12-h period.

Pro-inflammatory cytokines act as messengers from the local site of inflammation to trigger an acute phase response in the liver or other tissues. Activation of the acute phase response in the liver leads to changes in more than 200 circulating acute phase proteins (APP), which can be measured in blood as biomarkers of barrier dysfunction and endotoxin exposure. Acute-phase protein function varies—some act to limit microbial growth, others bind microbial fragments, neutralize enzymes, or scavenge free hemoglobin, and still others function to modulate the host’s immune response through negative feedback mechanisms. Elevated APPs are consistently observed in cattle exposed to subacute ruminal acidosis, heat stress, or aspirin-induced mucosal injury, making them practical indicators of leaky gut in both research and production settings. Lipopolysaccharide-binding protein (LBP) is a particularly useful indicator of gut barrier dysfunction because the gastrointestinal tract is the primary source of circulating LPS (Munford, 2016), and LBP concentrations have been positively associated with reductions in the jejunal villus height-to-crypt depth ratio (Kvidera et al., 2017b). Cytokine and APP are relatively easy to quantify with ELISA or clinical chemistry methods. These cytokine signals, whether arising from innate or adaptive activation, also contribute to systemic ­inflammatory responses. Although many cytokines and APP are not specific to the GI tract and may also rise during generalized stress, they are more directly linked to microbial product recognition and the TLR–NF-κB signaling cascade compared with immune cell counts, which also lack GI tract specificity. As such, they provide a more mechanistically informative indicator of luminal antigen exposure.

## Experimentally Inducing Gut Barrier Dysfunction

Understanding ruminant gut health requires models that reliably perturb the gastrointestinal tract (GIT) while allowing clear interpretation of downstream outcomes (permeability, inflammation, intake, performance). Broadly, two approaches are used: field-relevant stressors that mimic real-world production conditions and targeted pharmacological tools that focus more narrowly on epithelial biology.

Transportation is a widely used field model that exposes cattle to multiple stressors including fasting, dehydration, temperature change, and physical movement, which collectively disrupt gut barrier function, promote inflammatory responses, and decreases growth (Silva et al., 2023). Heat stress is another challenge with strong practical relevance. Elevated temperature and humidity reduce splanchnic blood flow, cause intestinal hypoxia, and promote tight junction disassembly, in addition to suppressing intake and altering hindgut fermentation (Koch et al., 2019). Feed restriction is also employed to model off-feed events and negative energy balance. By reducing substrate supply to the epithelium, this challenge alters villus–crypt dynamics and increases permeability. Pederzolli et al. (2018) reported that short-term feed restriction in beef steers decreased ruminal papillae size, and increased colon permeability. High-grain or subacute ruminal acidosis (SARA) challenges reproduce another common production stress by decreasing ruminal pH, shifting microbial communities, increasing endotoxin accumulation, increasing shedding of intestinal mucus, and damaging tight junctions and epithelia (Plaizier et al., 2012; Sanz-Fernandez et al., 2020). While all of these field-relevant models provide strong external validity, they perturb multiple physiological systems simultaneously, making it difficult to isolate gut-specific effects.

Targeted pharmacological models offer the benefit of acting directly on the GIT while disturbing fewer physiological systems. Nonsteroidal anti-inflammatory drugs (NSAIDs) such as aspirin and indomethacin reduce prostaglandin synthesis, impair mucus production, and decrease mucosal blood flow, all of which contribute to increased permeability (Klein et al., 2007; Briggs et al., 2020). Gamma-secretase inhibitor provides another targeted approach by blocking Notch signaling, which interferes with enterocyte maturation and compromises barrier integrity (Kvidera et al., 2017b). These compounds reduce villus height, increase crypt depth, and impair tight junction integrity, providing a clear epithelial biology signal. Although NSAIDs and gamma-secretase inhibitors reliably create permeability phenotypes, they do not mimic natural production stressors. Indomethacin and GSIs can also cause serious, dose-dependent gastrointestinal damage and are not approved for use in food-producing animals. Aspirin is comparatively less damaging to the gut, and until recently (2025) it was classified by the FDA as a compound of low regulatory concern, making it an attractive model since treated animals could still enter the food chain, reducing costs.

## Conclusions and Future Directions

Understanding and measuring gastrointestinal barrier function is essential for improving ruminant health and productivity. This requires integrating knowledge of rumen and intestinal physiology with methods that can detect structural, functional, and immunological changes in the epithelium. Because no single metric fully captures the complexity of barrier integrity, the best approach uses complementary measures.

Functional assays such as marker probe assays quantify epithelial leakiness, whereas structural assessments—including tight junction expression, villus height and crypt depth, and mucus integrity—provide mechanistic insight into epithelial damage and repair. Immunological biomarkers reflect the systemic consequences of luminal antigen translocation. When these measures converge, evidence for barrier dysfunction becomes far more compelling than any single measurement alone. Each approach, however, has limitations. Functional probe assays often require repeated sampling or catheterization, while molecular and histological methods rely on invasive tissue collection. Blood biomarkers are indirect, not always gut-specific, and individual variation can contribute to high standard errors. Non-invasive strategies, such as fecal biomarkers, show promise as practical indicators but remain largely unvalidated in ruminants.

Ruminant systems offer unique experimental opportunities to advance methodological development. Fistulated animals allow precise delivery of probes into specific gastrointestinal compartments and enable controlled infusion strategies that account for digesta passage dynamics, approaches that are far more difficult to implement in non-ruminants. Whole-animal calorimetry could also help quantify the energetic burden of immune activation in ruminants, linking epithelial barrier dysfunction with metabolic cost. In addition, techniques such as ex vivo tissue assays and intestinal organoids provide promising platforms to investigate epithelial barrier mechanisms and responses to luminal stressors under controlled conditions.

Progress towards improving ruminant gut health will depend on refining ruminant models, improving integration of complementary measurements, and developing minimally invasive, field-ready diagnostic tools. Improved permeability probes—including compounds that distinguish between tight junction pore transport, paracellular leak pathways, and unrestricted damage pathways—would further enhance interpretation of barrier function. In addition, many mechanistic insights regarding epithelial permeability, immune signaling, and tight junction regulation are derived from non-ruminant species. Confirming these mechanisms directly in ruminant models will be essential for ensuring that diagnostic tools and interventions are appropriately tailored to ruminant physiology.

Developing minimally invasive, field-ready tools would expand our ability to monitor ruminant gut health in real time and under commercial conditions. By combining existing techniques with new molecular and biomarker approaches, research can move toward practical applications that support cattle health, welfare, and productivity.

## About the Author

**Dr. Jon Schoonmaker** is a professor in the Department of Animal Sciences at Purdue University. Dr. Schoonmaker is originally from Wisconsin and received his bachelor’s degree from the University of Wisconsin-Madison. Dr. Schoonmaker received his master’s and doctorate degrees in beef cattle nutrition and management from The Ohio State University, where his research focused on growth composition and management strategies in early-weaned and conventionally weaned cattle. Following his graduate work, he spent time at the Carrington Research Extension Center at North Dakota State University as a researcher and later served as a technical consultant with Land O’Lakes Feed. He then spent 3 years at Iowa State University as a postdoctoral researcher studying the genetics of fatty acid composition in beef and milk. The goal of Dr. Schoonmaker’s research at Purdue is to create knowledge that will improve efficiency of growth for beef cattle through nutritional strategies that regulate gastrointestinal tract barrier function. Dr. Schoonmaker has led investigations to evaluate the use of feeds, nutrients and feed additives on ruminal fiber digestibility, acid-base status, nutrient availability, immune status, and the translational potential for beef cattle feeding management. Corresponding author: jschoonm@purdue.edu
